# Growth condition dependence of unintentional oxygen incorporation in epitaxial GaN

**DOI:** 10.1080/14686996.2016.1178565

**Published:** 2016-05-16

**Authors:** Felix Schubert, Steffen Wirth, Friederike Zimmermann, Johannes Heitmann, Thomas Mikolajick, Stefan Schmult

**Affiliations:** ^a^ Namlab gGmbH, Dresden, Germany; ^b^ Max-Planck-Institute for Chemical Physics of Solids, Dresden, Germany; ^c^ TU Bergakademie Freiberg, Institute of Applied Physics, Freiberg, Germany; ^d^ TU Dresden, Institute of Semiconductors and Microsystems, Dresden, Germany; ^e^ TU Dresden, Institute for Materials Science, Dresden, Germany

**Keywords:** MBE, GaN, oxygen incorporation, 40 Optical, magnetic and electronic device materials, 103 Composites, 100 Materials, 105 Low-Dimension (1D/2D) materials, 100 Materials, 201 Electronics / Semiconductor / TCOs, 200 Applications, 302 Crystallization / Heat treatment / Crystal growth, 300 Processing / Synthesis and Recycling, 501 Chemical analyses, 500 Characterization, 505 Optical / Molecular spectroscopy, 500 Characterization

## Abstract

Growth conditions have a tremendous impact on the unintentional background impurity concentration in gallium nitride (GaN) synthesized by molecular beam epitaxy and its resulting chemical and physical properties. In particular for oxygen identified as the dominant background impurity we demonstrate that under optimized growth stoichiometry the growth temperature is the key parameter to control its incorporation and that an increase by 55 °C leads to an oxygen reduction by one order of magnitude. Quantitatively this reduction and the resulting optical and electrical properties are analyzed by secondary ion mass spectroscopy, photoluminescence, capacitance versus voltage measurements, low temperature magneto-transport and parasitic current paths in lateral transistor test structures based on two-dimensional electron gases. At a growth temperature of 665 °C the residual charge carrier concentration is decreased to below 10^15^ cm^−3^, resulting in insulating behavior and thus making the material suitable for beyond state-of-the-art device applications.

## Graphical abstract

**Figure:**
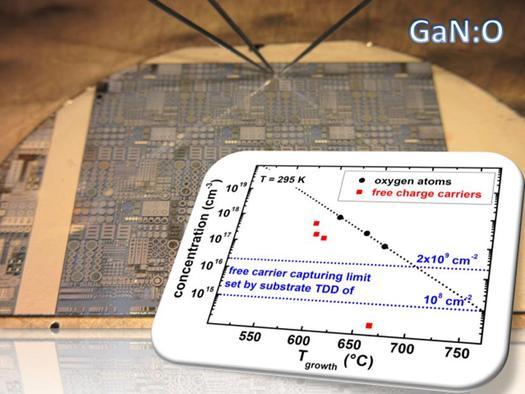

## 1. Introduction

Because of its large bandgap of 3.4 eV, gallium nitride (GaN) based electronics is a promising concept for high-frequency, high-power and white-light emitting devices. In recent years many investigations regarding the material properties of GaN and GaN-based heterostructures fabricated by various growth methods have been reported and summarized.[1] A key challenge for the fabrication of high-performance devices is still an uncontrollable background impurity level in GaN crystals. Compensating GaN with various elements is a legitimate way of obtaining insulating material. On the other hand the additional impurities will alter properties of e.g. high-electron mobility transistors (HEMTs) and diminish the superior performance expected from intrinsic (ultra-pure) material. As the history of the GaAs/AlGaAs system shows, the progress in growth techniques led to improvement in commercial devices [2] as well as discoveries of new fundamental physical effects in low-dimensional electronic systems.[3] The observation of the fractional quantum Hall effect as an example of electron–electron interaction physics was a result of mature sample growth by molecular beam epitaxy (MBE).[4] Despite innovative device concepts GaN based heterostructures also offer the possibility to probe different regimes in mesoscopic physics, due to the different material properties compared to GaAs.[5] Residual conductivity is often observed in GaN grown by MBE.[6,7] Oxygen has been identified as a main source of unintentional background impurities leading to n-type conductivity.[8]

This study confirms that oxygen is the predominant impurity in the investigated MBE-grown samples. More importantly it is shown that its incorporation can be controlled by adjusting the growth conditions properly. In particular, under perfect stoichiometric conditions the growth temperature (T_growth_) is the only parameter found to set the oxygen background in the epitaxial GaN. Within the growth temperature range 600–665 °C the GaN bulk free-carrier background concentration is reduced from 2 × 10^17^ cm^−3^ to below 10^15^ cm^−3^. Consistently, the photoluminescence (PL) intensity ratio of the free exciton and the donor-bound exciton peaks significantly increases. In GaN/AlGaN heterostructures hosting two-dimensional electron gases (2DEGs) an increase in mobility was observed at reduced oxygen background. Accordingly, HEMT structures based on these 2DEGs showed dramatically improved switching characteristics resulting in on-to-off source-drain-current ratios (I_on_/I_off_) up to 10^7^.

## 2. Experimental section

The samples investigated here were grown in a VG Semicon V80H MBE system in uninterrupted growth runs at constant substrate temperature measured by optical pyrometry. For all growth runs active nitrogen was supplied from an RF-plasma source operating at 250 W forward power fed with 0.6 sccm purified nitrogen (10 N) setting the growth rate to 250 nm h^–1^. Ultra-pure Ga (8 N) and Al (6N5) specimens were used which yield low-temperature electron mobilities in GaAs/AlGaAs heterostructures exceeding 10^7^ cm² V^–1^ s^–1^.[9] The samples were grown under slightly Ga-rich growth conditions resulting in atomically flat surface terraces and extremely sharp interfaces as well as homogeneous and precisely adjusted aluminum mole fraction in each grown layer for all investigated growth temperatures, as routinely investigated by atomic force microscopy and high-resolution X-ray diffraction. Under these outstanding growth conditions the substrate quality limits the structural perfection of the grown heterostructures.[10,11] The growth experiments were commonly performed on approximately 1.8 μm thick 2″ semi-insulating metal-polar hexagonal GaN templates prepared by metal organic chemical vapor deposition (MOCVD) on sapphire resulting in threading dislocation densities in a low- to mid-10^9^ cm^−2^ range as evaluated by X-ray diffraction measurements. These dislocations are known to capture free electrons from the bulk material in the low-10^16^ cm^−3^ range.[12] Several hundred nm thick GaN (bulk) as well as 2DEG structures consisting of 1 μm MBE-GaN buffer followed by 16 nm epitaxial Al_0.1_Ga_0.9_ N capped with 3 nm GaN were grown at different temperatures. This heterostructure design is known to yield a low sheet electron density around 2 × 10^12^ cm^−2^ [13] and was chosen to achieve a clear contrast between desired and undesired current paths in lateral transport measurements.

The free charge carrier concentration N_CV_ was profiled at room temperature by the coordinate transformation [14] of capacitance versus voltage (C(V)) measurements performed at 100 kHz with an Agilent B1505A Power Device Analyzer (Agilent Technologies Inc., Santa Clara, USA). The required Schottky-diode was fabricated via shadow mask based selective deposition of a Ti/Al/Ni/Pt ohmic contact stack annealed at 800 °C and platinum Schottky top electrodes with 120 μm in diameter. Pieces from the same wafer were used to fabricate laterally defined Hall-bars with channel length of 2.4 mm and width of 100 μm in a two-step ultraviolet lithography procedure for the mesa isolation (by dry-chemical etching) and ohmic contact definition. The Hall-bars were mounted into a DIL-socket for magneto-transport measurements performed in a physical property measurement system (PPMS) reaching temperatures down to 1.8 K and a magnetic field up to 9 T. Depth-dependent element concentrations in the grown samples were determined by dynamic secondary ion mass spectrometry (SIMS) with a depth resolution of 3 nm calibrated on element-specific GaN standards with a detection limit for oxygen in GaN of 5 × 10^16^ cm^−3^. Photoluminescence (PL) measurements were performed at 15 K with a continuous-wave helium-cadmium laser at 3.815 eV photon energy exciting the material above the absorption edge of GaN with a power density of 63 mW mm^–^². The luminescence signal was dispersed by a 0.5 m grating monochromator equipped with a 2400 lines mm^–1^ grating and detected with a liquid nitrogen cooled ultra-violet enhanced Si-CCD. The overall spectral resolution is limited to 1.25 meV.

## 3. Results and discussion

**Figure 1.**
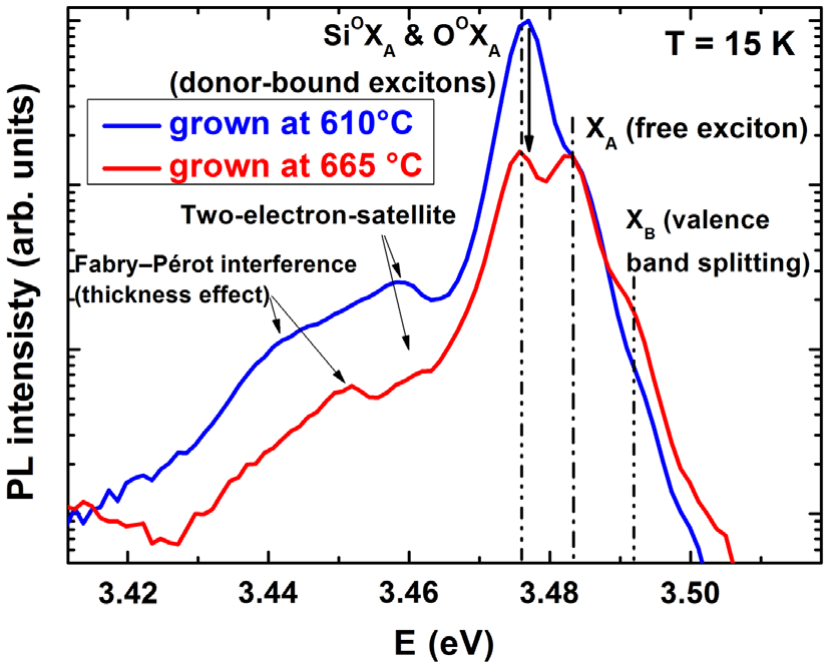
Photoluminescence spectra of GaN bulk samples grown at 610 and 665 °C recorded at 15 K and 63 mW mm^–^² of the 3.815 eV excitation laser light. The spectra are normalized to the GaN band gap (free exciton) PL intensity. A significant reduction in donor-bound exciton PL intensity is clearly visible for the higher growth temperature, as well as a pronounced peak attributed to the valence band splitting. The PL set-up resolution does not allow O and Si donors in GaN to be distinguished. The positions of two-electron satellites and Fabry-Pérot interferences are indicated.

**Figure 2.**
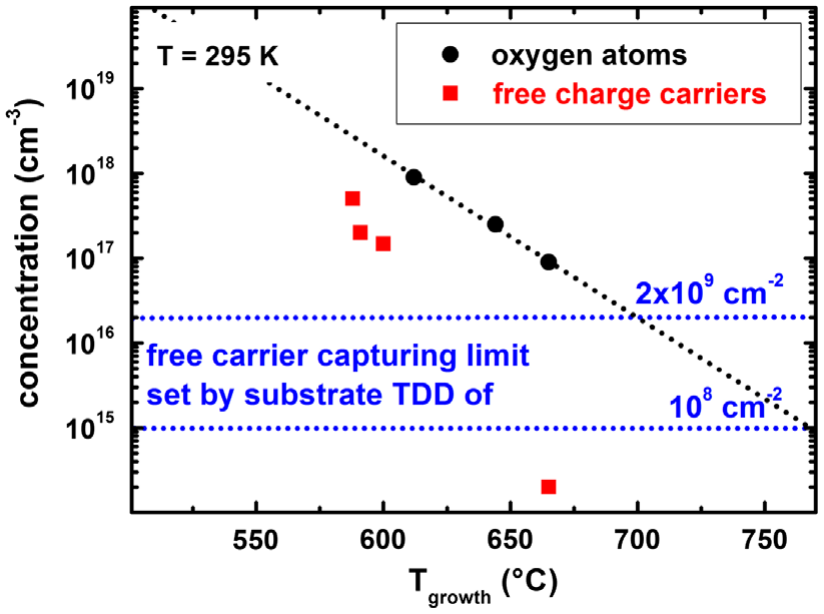
Unintentional oxygen background in GaN grown at temperatures between 610 and 665 °C (solid dots, the line is a guide to the eye). In this temperature range a significant reduction in oxygen concentration of one order of magnitude is detected. For comparison, the free carrier concentration determined by Hall-measurements and C(V) profiling is presented (solid squares). The capturing levels for free carriers by threading dislocations (see text) are shown for two threading dislocation densities (TDD) (dotted horizontal lines). Samples for 610 and 645 °C were not grown on semi-insulating substrates and thus a reliable determination of the free carrier concentration from electrical data was not possible due to parasitic current paths.

Low-temperature PL spectra of bulk GaN grown at 610  and 665 °C (Figure 1) show a significant decrease of the donor-bound exciton intensity at an energy position of 3.476 eV in relation to the free exciton peak at 3.483 eV with increasing growth temperature. This indicates a significant reduction in donor concentration. Quantitatively this reduction is verified by SIMS measurements of the incorporated background concentration of the elements oxygen, silicon, carbon, tantalum, manganese, arsenic and boron. However, for almost all of these elements the concentration is near or below the detection limit. Our results point out that only the detected oxygen concentration is changed in these samples. Figure 2 displays the oxygen concentrations in the two samples discussed in PL measurements and one GaN sample grown at intermediate (645 °C) temperature to manifest the trend.

**Figure 3.**
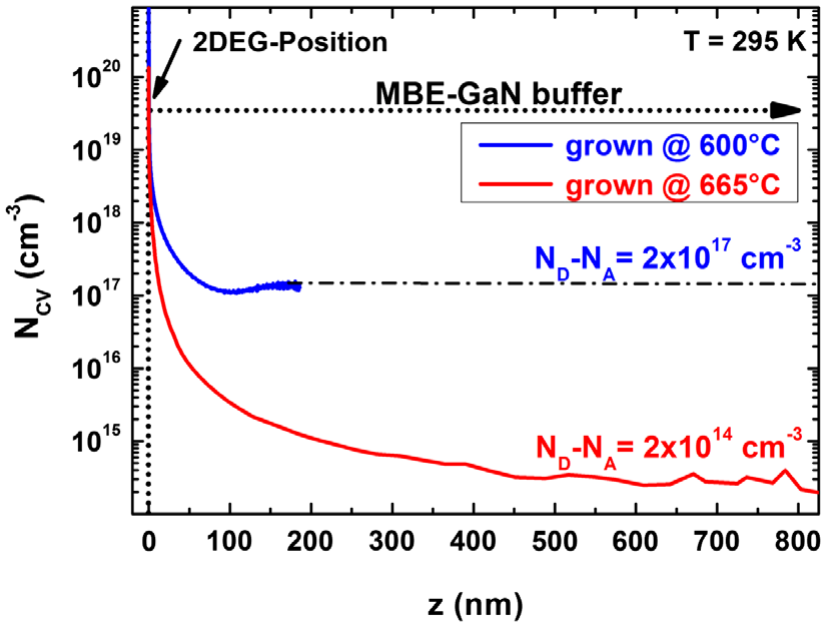
Carrier concentration profile extracted from C(V) measurements at 100 kHz of 2DEG structures grown at 600 and 665 °C. The 2DEG position is set to 0 nm. A significant reduction in the background free carrier concentration in the GaN buffer layer is observed for higher growth temperature.

Between 610 °C and 665 °C the oxygen background concentration reduces by one order of magnitude. In this temperature range the reaction equilibrium between the formation of Ga-O and Ga-N bonds is shifted exponentially. Simultaneously to the reduced oxygen background the concentration of free background electrons in GaN extracted from Hall and C(V) measurements is shown for samples grown on semi-insulating substrates. While at lower growth temperatures the determination of the density from Hall measurements is routine, this method fails for material grown at higher temperature due to the electrically insulating behavior. Here the C(V) method has a clear advantage and reveals simultaneously the negative charge character of the background carriers. Figure 3 shows free carrier distribution profiles extracted from C(V) measurements for 2DEG structures grown at 600 and 665 °C. For both samples the 2DEG position (set to 0 nm) is clearly visible by means of the high accumulated carrier density. The tail level represents the background free carrier concentration in the GaN buffer material and its value matches well with the Hall measurement results at low growth temperatures.

**Figure 4:**
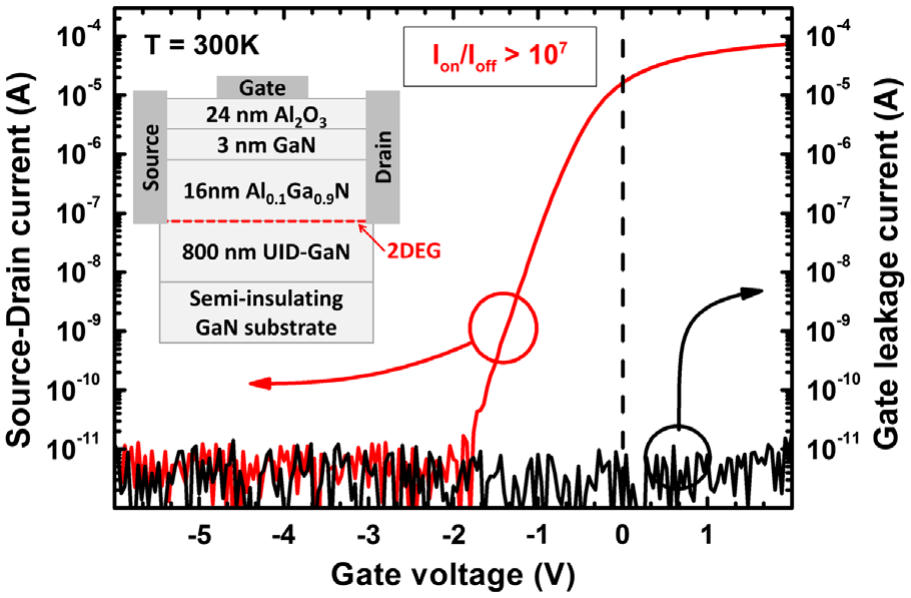
Transfer characteristic and gate leakage of a lateral field-effect transistor patterned on a 2DEG structure grown at 665 °C on semi-insulating GaN with gate width and length of 300 and 50 μm respectively. A cross-sectional device schematic is shown in the inset. The source-drain voltage was set to 100 mV, off-current and gate leakage are limited by the noise level of the measurement set-up and the on/off current ratio is > 10^7^.

Lateral field-effect transistor test structures based on these 2DEGs were evaluated by their switching characteristics, namely I_on_/I_off_. For the 2DEG grown at lower temperature I_on_/I_off_ was < 2 in comparison to 10^7^ for the structure grown at 665 °C (Figure 4). Thus in the case of a high background impurity level the transistor could not be switched off due to buffer leakage while in the case of an intrinsic buffer grown at higher temperature the transistors exhibited state-of-the-art transfer characteristics.

A significant decrease of almost three orders of magnitude in free carrier concentration in GaN down to below 10^15^ cm^−3^ at elevated growth temperature was found in contrast to only one order of magnitude for the oxygen background concentration; see Figure 2. However, similar trends are expected for the reduction of the free carrier concentration and for the oxygen concentration, since oxygen is known to act as a shallow donor in GaN with an activation energy of 29 meV.[15,16] Note that it becomes impossible to detect free background charges in the 10^16^ cm^−3^ range due to charge capturing by dislocations as discussed above. One remaining question concerns the origin of the oxygen. As a consequence of great care bestowed on the MBE system no traces of oxygen in the background vacuum at partial pressures > 10^−12^ Torr could be detected by mass spectrometry, exposing the source materials Ga and N as a plausible contamination source. In fact previous work points at the high-purity gallium as an inevitable source of oxygen.[9]

**Figure 5.**
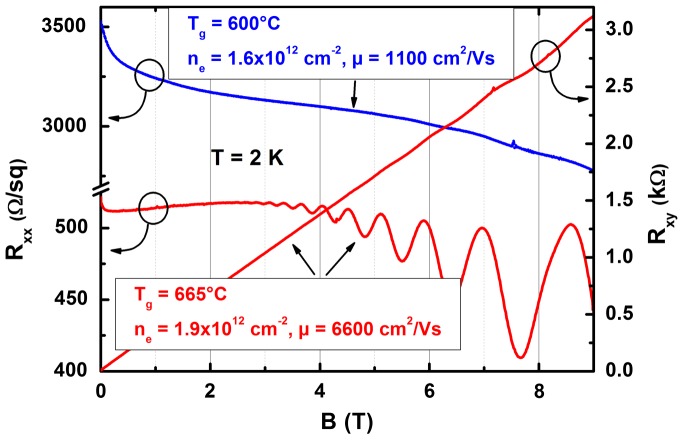
Magnetotransport data taken at 2 K for 2DEG structures grown at 600 and 665 °C. A significant increase in electron mobility is observed accompanied by pronounced Shubnikov–de Haas-oscillations in the sheet resistance for the sample grown at higher temperature. The Hall resistance of this sample shows signature of the quantum Hall effect at higher magnetic fields. The extracted densities from these traces result in identical values.

Oxygen, as the predominant donor in the samples studied, represents a charged impurity that will alter transport properties of 2DEGs. Quantum transport at low temperatures is known to be very sensitive to the background impurity level.[3] Figure 5 displays magnetic field-dependent resistances at 2 K of 2DEG samples grown at different temperatures. The 2DEG grown at 665 °C exhibits a substrate dislocation density-limited low-temperature mobility [17,18] of 6600 cm² V^–1^ s^–1^ and well pronounced Shubnikov–de Haas oscillations in the longitudinal resistance commencing at 2.4 T. Identical electron densities of 1.9 × 10^12^ cm^−2^ were determined in room-temperature Hall measurements and in the quantum transport regime from three independent methods: the low-field Hall coefficient; the Shubnikov–de Haas oscillation frequency; and the appearing quantum Hall resistance plateaus, demonstrating that the 2DEG is the only conductive channel. The resulting value of the 2DEG density is expected for this particular heterostructure design. In contrast, the 2DEG structure grown at 600 °C does not show signatures of quantum transport. At 2 K, for this sample a 2DEG density of 1.6 × 10^12^ cm^−2^ and a corresponding carrier mobility of ≈1100 cm² V^–1^ s^–1^ was extracted from the Hall coefficient. The comparison of the nominally identical samples clearly manifests that the altered transport properties originate from the impurity scattering caused by the enhanced oxygen incorporation at lower growth temperature.

**Figure 6.**
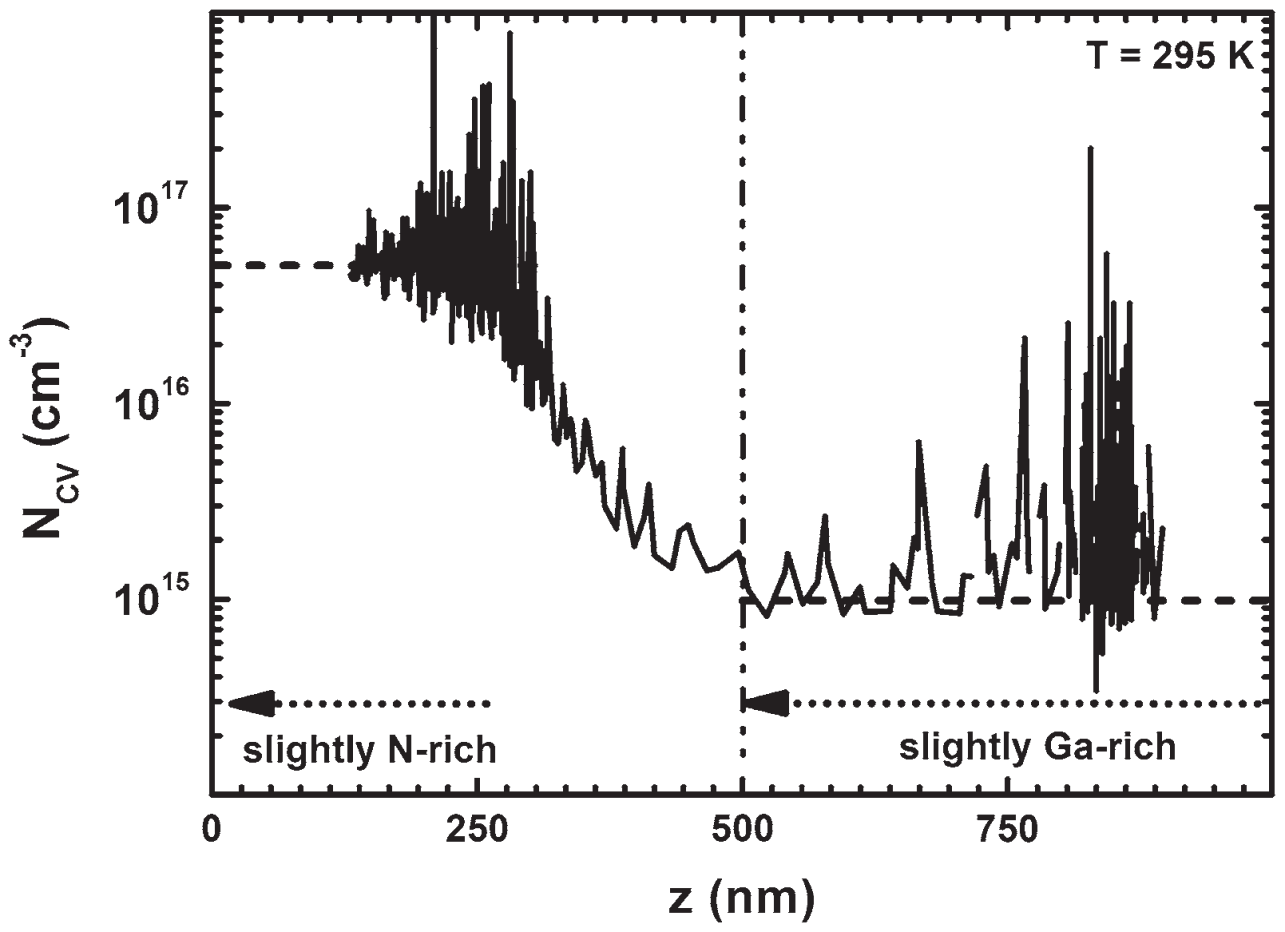
Carrier concentration profile extracted from C(V) measurements of a 1 μm thick GaN layer consisting of 500 nm GaN grown under slightly Ga-rich and 500 nm GaN grown under slightly N-rich conditions at 665 °C. The stabilized average carrier concentration level in the N-rich grown GaN is 5 × 10^16^ cm^−3^ while it is below 1 × 10^15^ cm^−3^ in the GaN grown under Ga-rich conditions indicated by dashed lines.

As the amount of incorporated oxygen was correlated with the growth temperature under identical stoichiometric growth conditions, further attention was paid to the influence of the stoichiometry at constant growth temperature. Figure 6 shows the free carrier concentration profile of a 1 μm thick GaN layer uninterruptedly grown at 665 °C. After 500 nm the slightly Ga-rich growth conditions were changed to slightly N-rich conditions by reducing the Ga-flux by 2%, resulting in an increase of the free background charge carrier concentration from below 10^15^ cm^−3^ to 5 × 10^16^ cm^−3^. This result unambiguously indicates that not only the growth temperature but also the growth stoichiometry has tremendous impact on the oxygen incorporation in MBE-grown GaN.

## 4. Conclusions

In summary, various characterization methods contribute to a consistent picture and expose oxygen as the dominant unintentional donor-type dopant in GaN grown by plasma-assisted MBE. For optimized III/V growth stoichiometry the growth temperature is the key parameter to control the oxygen incorporation. An oxygen level reduction by one order of magnitude was achieved for an increase of growth temperature by only 55 °C and can be eventually eliminated undetectably for higher growth temperature. In consequence the background free carrier concentration as well as the donor-bound exciton peak intensity in PL spectra drastically decreased. This consistently resulted in higher electron mobilities in 2DEG heterostructures and undetectably low buffer leakage currents in lateral field-effect transistors.

## Disclosure statement

No potential conflict of interest was reported by the authors.

## Funding

This work was financially supported by the German Federal Ministry of Education and Research (BMBF) [project 16ES0145 K] and by the EFRE fund of the European Community and by funding from the Free State of Saxony [project: GaNFET, number 2042/1949].
